# Factors associated with poor compliance amongst hospitalized, predominantly adolescent pediatric Crohn’s disease patients

**DOI:** 10.1080/07853890.2022.2057582

**Published:** 2022-03-30

**Authors:** Nathaniel A. Cohen, Dejan M. Micic, Atsushi Sakuraba

**Affiliations:** Inflammatory Bowel Disease Center, University of Chicago Medicine, Chicago, IL, USA

**Keywords:** Compliance, paediatric gastroenterology, Crohn’s disease, inflammatory bowel disease

## Abstract

**Background:**

Compliance with medical treatment is vital for the control of inflammatory bowel disease (IBD) and prevention of disease complications and is an issue in paediatric patients. We explored patient-related factors associated with non-compliance in a large database of predominantly adolescent, hospitalized paediatric Crohn’s disease (CD) patients.

**Patients/Materials and Methods:**

We analyzed data from the Kid’s Inpatient Database (KID) the largest publicly available all-payer paediatric inpatient care database in the United States. All available paediatric CD patients non-electively admitted in 2016 were included. CD patients were extracted using the standard international classification of diseases (ICD) 10 codes. Data suggesting non-compliance, comorbidities and surgical procedures related to CD were similarly extracted.

**Results:**

2439 paediatric CD patients with non-elective admission were included in the analysis. 2 280 patients (94%) were adolescents. Of the total cohort, 113 patients (4.6%) had a diagnosis of non-compliance. In univariate analyses, smoking (15.9 vs. 5.5%, *p* < .001), cannabis use (5.3 vs 1.5%, *p* = .009), and a diagnosis of depression (19.5 vs. 9%, *p* = .001) or schizoaffective disorder (5.3 vs 0.3%, *p* < .001) were associated with non-compliance. Multivariable analysis revealed that schizoaffective disorder (odds ratio (OR) 11.6, 95% CI 3.6–37.2), depression (OR 1.9, 95%CI 1.2-3.2) and smoking (OR 2.2, 95%CI 1.25–4) were independently associated with non-compliance.

**Conclusions:**

In this study, mental health disorders and smoking were independently associated with non-compliance to medication in predominantly adolescent, hospitalized paediatric CD patients. A multidisciplinary approach involving paediatric gastroenterologists, psychiatrists and addiction specialists are needed to treat the underlying causes and improve adherence in these patients.

**KEY MESSAGES**
- Mental health disorders and smoking are independent risk factors for medication non-compliance amongst adolescent, paediatric CD patients.
- A multidisciplinary approach is required to treat underlying causes and improve adherence in paediatric IBD patients.

## Introduction

Inflammatory bowel diseases (IBD), comprising Crohn’s disease (CD) and ulcerative colitis (UC), are chronic, inflammatory conditions which primarily affect the gastrointestinal tract. Incompletely controlled disease may lead to irreversible bowel damage including fibrosis with resulting strictures, fistulae and cancer [1]. These all adversely impact the quality of life of patients with IBD and often result in the need for multiple hospitalizations and surgeries [2]. Treatment of IBD requires the chronic use of immune-modulating and immunosuppressive maintenance medications in order to control inflammation and prevent complications [3]. As with all chronic medications, adherence to the prescribed treatment plan remains a challenge [4].

IBD can affect people of any age. However, around 25% of IBD patients are diagnosed during childhood or adolescence [5]. Adherence to medication regimens is critical to their long-term success regardless of age. Indeed, poor adherence has been associated with increased disease activity, relapse, loss of response to anti-tumor necrosis factor (TNF) agents, increased health expenditure, poor quality of life and increased disability [6–10].

Paediatric populations pose significant challenges regarding adherence to medical care. Prior studies have demonstrated non adherence rates of up to 66% in the paediatric population [11]. The reasons are multifactorial and can be divided into disease-related, patient-related, medication-related, parental- and physician-related or systematic factors. Disease-related factors include the relapsing and recurring nature of IBD, ongoing inflammation in the setting of minimal clinical symptoms, requirement for multiple clinical visits, hospitalizations and surgery. Patient-related factors include behavioural and developmental functioning. Medication-related factors include complicated treatment regimens with multiple pills or requirements to take multiple times a day as well as drugs with intravenous or intramuscular administration. Parental factors include education level, financial and insurance situation. Lastly, physician-related or systematic factors include poor education of and communication with paediatric patients and their parents as well as access to adequate, timely and consistent care.

In most cases, the aforementioned factors, likely combine to make treatment adherence a challenge for both patients and their families. The aim of this study was to use a large database of hospitalized paediatric CD patients in order to identify patient-centered risk factors for non-compliance in this patient population.

## Methods

### Study design and data source

We performed a cross-sectional analysis using the 2016 Kid’s Inpatient Database (KID), a nationally representative sample of paediatric hospitalizations from the Healthcare Cost and Utilization Project (HCUP), sponsored by the Agency for Healthcare Research and Quality (AHRQ). KID is the largest all payer paediatric inpatient database from the United States and includes around 3 million hospitalizations in the year 2016 [12]. The database contains over 75 clinical and non-clinical variables, diagnoses and procedures encoded using the *International Classification of Diseases,* 10th Edition (ICD-10) in addition to hospital characteristics [12].

### Study sample

We examined discharge data from hospitalizations of paediatric patients age 20 years or younger. We defined adolescence as patients between the ages of 10 and 24 as previously described by Sawyer et al [13]. Hospitalizations with an ICD-10 code indicating CD (K50.X) at position #1 (primary diagnosis) or position #2 (secondary diagnosis) were included. Similar inclusion rules have been used previously [14–16]. We excluded hospitalizations which were recorded as elective in order to investigate non-compliance specifically in patients presenting for urgent/emergent admission and increase the likelihood of capturing patients with active/poorly controlled disease.

### Outcome variable

The outcome variable of interest was any diagnosis of “noncompliance” which was identified using the ICD-10 diagnosis codes: “Z91.19, Z91.14, Z91.11, Z91.128, Z91.120”. These codes all represent non-compliance with medical treatments and regimens.

### Other variables

Data collected from each paediatric hospitalization included patient age, sex, race (White, Black, Hispanic, Asian, Native American and other), payer type (Medicare, Medicaid, private insurance, self-pay, no charge and other), patient location (central metropolis, fringe metropolis, county with a population between 250 and 999K, county with a population between 50 and 250K and micropolis), ZIP code income status divided into quartiles with the first quartile being the lowest income and fourth quartile the highest. Other diagnosis and surgical procedures related to CD were identified using ICD-10 codes. The complete set of ICD codes used for the present study are shown in Supplementary Table 1.

### Statistical analysis

All statistical analysis was preformed using SPSS (IBM corporation, version 26). Continuous variables with a normal distribution were compared using the student’s *t*-test and those with non-parametric distribution were compared using the Mann-*U*-Whitney test. Categorical variables were compared using the *X*^2^ or Fisher’s exact test where appropriate. Multivariable logistic regression was performed to determine associations between the predictor variables (with a significance <0.1), age and sex and a diagnosis of non-compliance. The *p* value was considered significant at a level of .05 or less.

## Results

**Figure 1.**
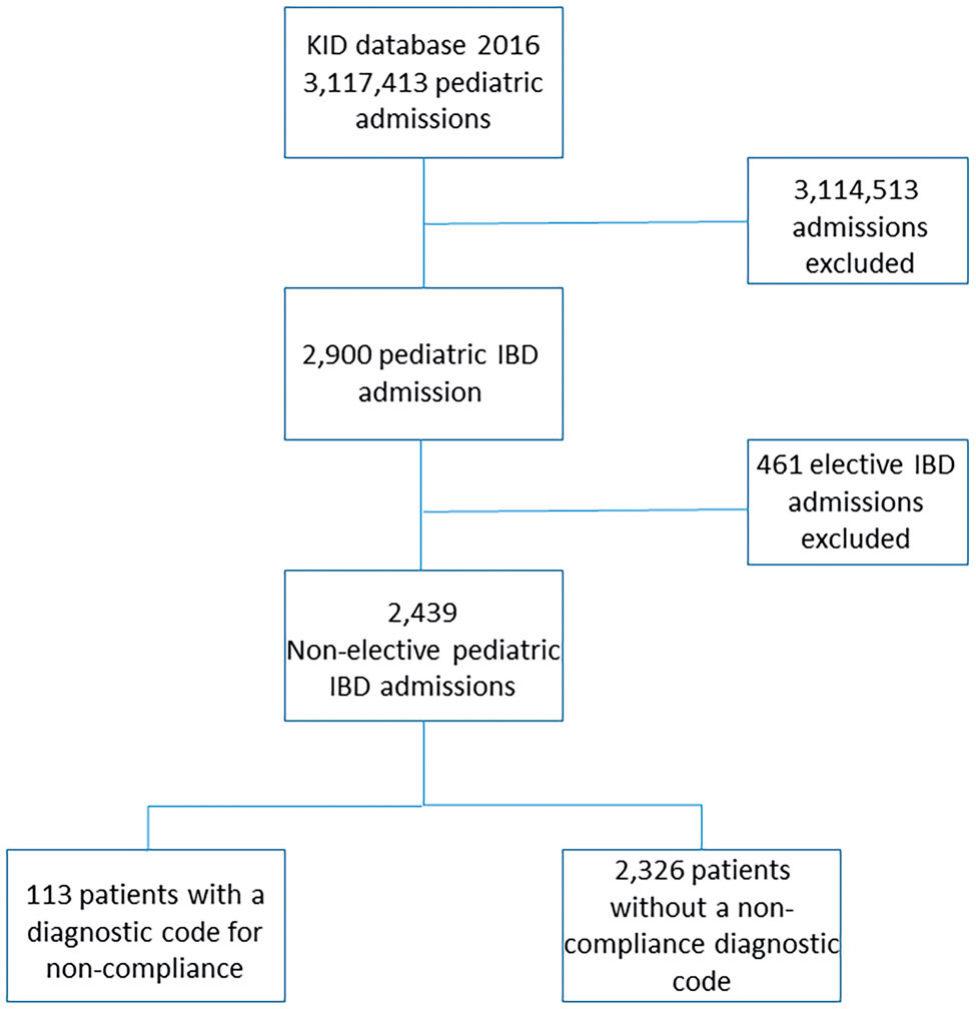
Flow chart detailing the study design.

The KID of 2016 included 3,117,413 paediatric hospitalizations, of which 2,900 hospitalizations had a primary or secondary diagnosis of CD. 461 patients had elective admissions which were excluded, leaving 2,439 CD hospitalizations in the analysis for this study (Figure 1). A total of 113 patients had an ICD-10 code for a diagnosis of non-compliance and these patients were compared to the 2,326 patients without such diagnosis (control cohort).

### General demographics and disease characteristics

The median age of the non-compliant and the control cohorts was similar (17 (interquartile range (IQR) 13–19) vs. 17 (IQR 14–19), *p* = .46; respectively). Of note, 2280 of the included patients (94%) were in the adolescent age range. There were also similar sex distributions in both non-compliant and control cohorts with females comprising 49.6% and 47.5% (*p* = 0.7) of the cohorts, respectively. There were no significant differences in terms of race, insurance payer, patient location or ZIP code income status. Further demographic details are represented in Table 1. There was no significant difference in disease location between the cohorts (Table 1). There were no significant differences in terms of length of hospitalizations and hospital charges between the groups (median 3 (IQR 2–6) vs 3 (IQR 2–6), *p* = .52 and median $29,137 (IQR 16532–53293.75) vs. $28,146.5 (IQR 17997 − 48057.5), *p* = .98; respectively).

**Table 1.** Demographic details of the entire patient cohort.

Variable	Non-compliance reported (*n* = 113)	Non-compliance not reported (*n* = 2326)	*p*-Value
Age - median (IQR)	17 (13-19)	17 (14-19)	.46
Female – *n* (%)	56 (49.6)	1106 (47.5)	.7
CD location			
Ileal – *n* (%)	19 (16.8)	567 (24.4)	.07
Colonic – *n* (%)	18 (15.9)	333 (14.3)	.59
Ileocolonic – *n* (%)	21 (18.6)	446 (19.2)	.7
Indeterminate IBD – *n* (%)	4 (3.5)	105 (4.5)	.82
ZIP income status			
1st Quartile – *n* (%)	22 (19.5)	451 (19.4)	1
4th Quartile – *n* (%)	40 (35.3)	767 (33)	.6
Race			
White - *n* (%)	78 (69%)	1587 (68.2)	.92
Black - *n* (%)	14 (12.4)	324 (13.9)	.78
Hispanic – *n* (%)	10 (8.8)	134 (5.8)	.22
Asian – *n* (%)	2 (1.8)	39 (1.7)	.72
Native American – *n* (%)	1 (0.9)	11 (0.5)	.44
Other – *n* (%)	6 (5.3)	115 (4.9)	.82
Payer			
Medicare – *n* (%)	0(0)	10 (0.4)	1
Medicaid – *n* (%)	39 (34.5)	743 (31.9)	.6
Private insurance – *n* (%)	71 (62.8)	1484 (63.8)	.84
Self-pay – *n* (%)	1 (0.9)	40 (1.7)	1
No charge – *n* (%)	1 (0.9)	4 (0.2)	.2
Other – *n* (%)	0 (0)	41 (1.8)	.26
Patient location			
Central metropolis – *n* (%)	26 (23)	627 (27)	.4
Fringe metropolis – *n* (%)	39 (34.5)	852 (36.6)	.69
County 250–999K population – *n* (%)	27 (23.9)	426 (18.3)	.14
County 50–250K population – *n* (%)	7 (6.2)	192 (8.3)	.6
Micropolis – *n* (%)	10 (8.8)	151 (6.5)	.33

IQR: interquartile range; CD: Crohn’s disease; *n*: number; IBD: inflammatory bowel disease.

### Substance use and non-compliance

The non-compliant cohort had significantly more diagnoses related to smoking (15.9 vs 5.5%, *p* < .001) and cannabis use (5.3 vs 1.5%, *p* = .009) compared with the control cohort. There was a non-significant trend towards greater opioid-use related diagnoses in the non-compliant cohort (1.8 vs 0.9%, *p* = .26).

### Mental health diagnosis and non-compliance

Overall, there was a significantly greater number of any mental health diagnosis in the non-compliant cohort compared with the control cohort (38 (33.6%) vs. 444 (19.1%), *p* < .001). Looking at specific mental health diagnoses, there was a significantly greater number of patients with a diagnosis of depression (19.5 vs. 9%, *p* = .001) and schizoaffective disorder (5.3 vs. 0.3%, *p* < .001) in the non-compliant cohort. There were no significant differences in number of patients with a diagnosis of anxiety, attention deficit and hyperactivity disorder, bipolar mood disorder or autism between the cohorts.

### Disease related complications and non-compliance

In this study there were no significant differences in rates of surgery between the cohorts nor were there significant differences in concurrent diagnoses of common CD related complications such as perianal abscesses, intra-abdominal and retroperitoneal abscesses, perforations or bowel obstructions. Furthermore, the use of steroids was similar between the cohorts (Table 2).

**Table 2.** Differences in multiple variables in the non-compliant and control cohort.

Variable	Non-compliance reported (*n* = 113)	Non-compliance not reported (*n* = 2326)	*p*-Value
Opioid use – *n* (%)	2 (1.8)	21 (0.9)	.29
Steroid use – *n* (%)	11 (9.7)	158 (6.8)	.25
Cannabis use – *n* (%)	6 (5.3)	34 (1.5)	.009
Smoking – *n* (%)	18 (15.9)	129 (5.5)	<.001
Mental health diagnosis			
Any mental health diagnosis – *n* (%)	38 (33.6)	444 (19.1)	<.001
Anxiety – *n* (%)	17 (15)	258 (11.1)	.22
ADHD – *n* (%)	9 (8)	102 (4.4)	.1
Depression – *n* (%)	22 (19.5)	209 (9)	.001
Bipolar mood disorder – *n* (%)	2 (1.8)	33 (1.4)	.7
Schizoaffective disorder – *n* (%)	6 (5.3)	7 (0.3)	<.001
Autism – *n* (%)	1 (0.9)	50 (2.1)	.73
Undergoing surgery – *n* (%)	20 (17.7)	353 (15.2)	.5
Disease complication			
Perforation – *n* (%)	4 (3.5)	68 (2.9)	.58
Intestinal obstruction – *n* (%)	4 (3.5)	111 (4.8)	.82
Perianal abscess – *n* (%)	7 (6.2)	120 (5.2)	.66
Peritoneal abscess – n (%)	1 (0.9)	57 (2.5)	.5
Psoas abscess – *n* (%)	0 (0)	13 (0.6)	1
Retroperitoneal abscess – *n* (%)	0 (0)	2 (0.1)	1

### Multivariable analysis looking at factors associated with non-compliance

**Figure 2.**
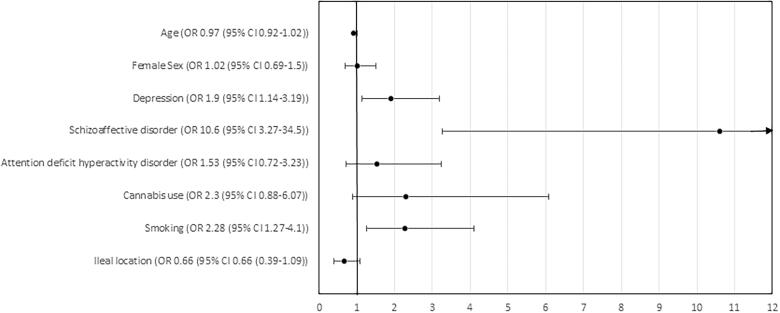
Forest plot showing the odds ratios of various factors associated with a diagnosis of non-compliance in hospitalized paediatric patients. OR: odds ratio; CI: confidence interval.

In a logistic regression analysis including variables with a significance of <0.1 and correcting for age and sex, patients with a diagnosis of schizoaffective disorder (odds ratio (OR) 10.6; 95% confidence interval (CI) 3.3–34.5), depression (OR 1.9; 95%CI 1.1–3.2) and a diagnosis indicating smoking use (OR 2.3; 95%CI 1.3–4.1) were all significantly and independently associated with a diagnosis of non-compliance (Figure 2).

## Discussion

Non-compliance to medical therapy and regimens affects the ability to control inflammation in IBD with ramifications including disease complications and decreased quality of life. Paediatric patients pose a unique challenge to compliance with medical therapies owing to the influence of a multitude factors. This study looked at patient and systemic factors associated with a diagnosis of non-compliance in a nationally representative cohort of predominantly adolescent, hospitalized paediatric CD patients in the US and found that depression, schizoaffective disorder and smoking were independently associated with non-compliance.

In the adult population, mental health conditions such as depression and anxiety have been shown to be associated with poor compliance to medical treatment. Indeed, in the general population those with depression are three times more likely to have poor compliance than non-depressed counterparts [17]. Depression is a significant issue in the paediatric IBD patient population with rates approaching 20% [18,19]. In our study we show that both depression and schizoaffective disorders are associated with paediatric non-compliance. It is thought that this association may be related to the impaired cognition, energy, motivation and willingness to follow medical instructions inherent in these conditions [17]. This study highlights the need for effective screening for depression and other mental illnesses in paediatric populations in order to help effectively and holistically manage IBD patient care, diagnose and treat mental illness and hopefully improve medication compliance.

Smoking in the paediatric and adolescent population is associated with multiple psychosocial problems. These include a lower belief in conventional rules and poor school performance [20,21], increased rates of risk taking behaviour [22,23], stress and depression [24,25], peer and family influence [26,27] and self-esteem issues [28]. As such, smoking tobacco as well as cannabis, which were both significantly increased in non-compliant patients, could potentially be seen as a marker for underlying biopsychosocial factors resulting in an increased risk of medical non-compliance and should raise a red flag in the mind of the treating physician leading to more focussed questioning relating to compliance as well as implementation behaviour modifications. Both mental illness and smoking are very prevalent in the IBD paediatric population primarily in adolescence [19,29,30]. As almost 95% of the patient population in this study were adolescent, these findings, are particularly relevant for healthcare providers treating these patients.

There are several limitations to this study. We report the association between an ICD-10 code for non-compliance and mental illness and substance use, however, no causal relationship can be determined. As this is a large database study with diagnoses and procedures encoded using ICD-10 codes, it is possible that certain variables may have been missed, under- or over-represented, in particular, disease activity and parental- or provider- related factors could not be assessed. These may all be significant confounding variables in such an analysis. The database depends on the correct reporting of diagnoses and procedures during an admission which may be limited or misrepresented, an issue which has been raised previously [31]. In particular, the diagnosis of non-compliance may not be clear in many instances thus leading to underreporting. Furthermore, due to the complex nature of non-compliance and the lack of granularity describing the reason for the presence of these ICD-10 codes, greater insight into this relationship cannot be determined but should be the focus of future studies. Despite these limitations such codes have been previously used in database studies investigating the effects of a diagnosis of medical non-compliance and therapy practices in patients with mental illness as well as outcomes in non-compliant nephrology patients [32,33]. It is also not possible to determine exact reason for admission and current medications. Another limitation is that this database is not reported on an individual basis thus it is not possible to track repeated hospitalizations or surgeries and longitudinal outcomes. The major strength of this study is that the KID is a very large multi-payer and nationally representative database meaning that these results are probably generalizable across the adolescent paediatric IBD population in the US. Another strength is that the methods of this study also replicate other studies which used ICD codes to identify various outcomes [16–18].

In conclusion, this study demonstrates that mental health disorders and smoking are independently associated with non-compliance in a predominantly adolescent cohort of hospitalized paediatric CD patients. The results of our study should raise the awareness of the treating physician to these and other related conditions which may negatively impact disease outcomes both in the short and long-term. A multidisciplinary approach including paediatric IBD specialists, psychiatrists and addiction specialists is required in order to tackle this issue holistically and improve adherence to medical therapy.

## Supplementary Material

Supplemental Material

## Data Availability

Data use in the project is available from the Healthcare Cost and Utilisation Project (HCUP).

## Abbreviations

IBD: inflammatory bowel disease
CD: Crohn’s disease
KID: Kid’s Inpatient Database
ICD: International Classification of Diseases
